# Highly efficient transgenesis mediated by *Tip100* transposon system in medaka

**DOI:** 10.1007/s11248-025-00466-5

**Published:** 2025-10-09

**Authors:** Yoshitaka Tanaka, Takahide Seki, Atsushi Hoshino, Satoshi Ansai

**Affiliations:** 1 Ushimado Marine Institute (UMI), Okayama University, Setouchi, Okayama 701-4303 Japan; 2 Laboratory of Genome Editing Breeding, Graduate School of Agriculture, Kyoto University, Kyoto, 606-8507 Japan; 3 Department of Integrative Life Sciences, Graduate School of Life Sciences, Tohoku University, Sendai, Miyagi 980-8577 Japan; 4 National Institute for Basic Biology, Okazaki, Aichi 444-8585 Japan; 5 Graduate Institute for Advanced Studies, SOKENDAI, Okazaki, Aichi 444-8585 Japan

**Keywords:** Fish, Medaka, Morning glory, Transgenic, Transposon

## Abstract

Transgenesis mediated by transposon is an effective approach for introducing exogenous DNA into the nuclear genome and establishing stable transgenic strains that efficiently express genetic tools. Although the DNA transposon *Tol2* is widely used for transgenesis in zebrafish, its endogenous transpositional activity can lead to unintended transgene mobilization, making it unsuitable for transgenesis in medaka (*Oryzias latipes*). Here, we demonstrated that the DNA transposon *Tip100*, originally identified in the common morning glory (*Ipomoea purpurea*), an ornamental plant, can serve as a useful tool for transgenesis in Japanese medaka. The GFP transgene cassette, when co-injected with *Tip100* transposase mRNA, was expressed in significantly higher number of somatic cells in the injected fish. Furthermore, a transgene flanked by truncated recognition sequences (100 bp each) exhibited expression levels comparable to those of the original vector containing the full 2.2 kb recognition sequence. Injection of a transgene driven by a germline-specific promoter revealed that fish injected with *Tip100* mRNA exhibited a significantly higher germline transmission rate (42/68; 62.7%) compared to those injected without the mRNA (13/62; 21.0%). We successfully established transgenic strains by outcrossing injected founders with GFP-positive germ cells (7/7; 100%) and demonstrated that the transgenes were randomly integrated into the medaka genome, generating 8-bp duplications at the insertional sites–an insertional signature of the hAT superfamily of transposons. Our findings indicate that the *Tip100* system is a promising tool for generating stable transgenic strains that express various genetic tools in medaka and potentially other fish species.

**Supplementary Information:**

The online version contains supplementary material available at 10.1007/s11248-025-00466-5.

## Introduction

The Japanese medaka (*Oryzias latipes*) is a small teleost fish that serves as a vertebrate model for genetics, alongside zebrafish (*Danio rerio*). Although these small fishes share several biological characteristics, medaka possesses additional advantages, such as the availability of diverse wild-derived strains and related species, high tolerance to temperature changes, and a robust genetic sex determination system (Takeda and Shimada 2010). Similar to zebrafish, recent advances in genetic engineering have enabled targeted gene modifications and the visualization of specific cells, facilitating the functional analysis of various biological processes in medaka (Kirchmaier et al. 2015).

Transgenesis, the integration of exogenous DNA fragments into genomes of organisms, is a straight-forward approach for analyzing the function of target genes and cells. Among various methodologies used to enhance genome integration efficiency, transposon-mediated transgenesis is a powerful method for introducing exogenous DNA. These systems typically consist of a DNA transposon vector and a transposase expression vector, which together mediate the excision of a transgene cassette flanked by inverted repeats and its subsequent genomic integration. This approach enables transgene insertion at random genomic positions with high efficiency and is widely used to establish transgenic lines in various organisms and cell types (Ivics et al. 2009). In zebrafish, the DNA transposon *Tol2* facilitates highly efficient genomic integration of DNA vectors and has been employed to generate numerous transgenic strains (Kawakami 2005), including gene/enhancer trap lines (Kawakami et al. 2004; Asakawa et al. 2008), which serve as essential tools for functional analysis. The *Tol2* system has been used for transgenesis not only in zebrafish but also in various other teleost species (Fujimura and Kocher 2011; Valenzano et al. 2011; Juntti et al. 2013; Erickson et al. 2016; Higaki et al. 2021; Miyamoto et al. 2024). However, because the *Tol2* element was originally identified as an active transposable element in the medaka genome (Koga et al. 1996), it has been shown to transpose in both somatic and germline cells (Iida et al. 2005; Koga et al. 2006), raising concerns that transgenes integrated via the *Tol2* system may be unstable in medaka. To address this limitation, alternative transposon systems such as *Sleeping Beauty* (Grabher et al. 2003) and maize *Ac/Ds* (Boon Ng and Gong 2011; Froschauer et al. 2012) have been applied to medaka transgenesis. However, their usage remains limited due to inconsistent efficiencies and the lack of publicly available plasmid resources. More recently, additional transposons such as *ZB* and *Passer* have been identified as potential genetic tools in fish genomes (Shen et al. 2021; Wang et al. 2023), but they have not yet been tested in medaka.

Here, we demonstrate that the *Tip100* DNA transposon can serve as an efficient tool for transposon-mediated transgenesis in medaka. *Tip100*, a member of the hobo-Ac-Tam3 (hAT) superfamily alongside *Tol2* and *Ac/Ds*, was originally identified at a mutable locus responsible for flower variegation in the common morning glory (*Ipomoea purpurea*), a climbing plant widely cultivated worldwide for its colorful flowers (Habu et al. 1998). A 3.8-kb functional copy of *Tip100* at this mutable allele contains 11-bp terminal inverted repeats (CAGGGGCGGAG) and a 2,427-bp open reading frame encoding an 808 amino acids of transposase (Habu et al. 1998) (Figure S1). Previous work showed that this transposase mediates autonomous transposition of *Tip100* in transgenic tobacco plants (Ishikawa et al. 2002), but the system has not been evaluated as transgenic tools in other species. Given its strong transpositional activity observed in variegated morning glory flowers and the generally high activity of hAT transposons in medaka, we hypothesized that the *Tip100* system would exhibit similarly high activity in medaka. First, we examined whether the *Tip100* transposon system could facilitate the genomic integration of a transgene cassette from a DNA vector in medaka embryos. Next, to enhance the usability of the *Tip100* system across various plasmid vectors, we optimized the length of its transposon recognition sequences including the inverted repeats. Finally, we assessed the efficiency of *Tip100*-mediated germline transgene integration and investigated whether transgene insertions occurred in a transposon-dependent manner.

## Materials and methods

### Fish husbandry

All medaka fish used in this study were maintained under a photoperiod of 14 h light/10 h dark and a water temperature of 26–28 °C in recirculating systems. The fish were fed three times per day with live nauplii (*Artemia* sp.) and commercial pellet food (Otohime; Marubeni Nisshin Feed). The himedaka strain (ID: MT835) of Japanese medaka (*Oryzias latipes*) was obtained from the National BioResource Project (NBRP) Medaka (https://shigen.nig.ac.jp/medaka) and used as the parental strain in this study. All procedures were conducted in accordance with the guidelines and regulations of the Animal Care and Use Committee of the Kyoto University (Approval number: R5-102).

### Preparation of plasmid DNA vectors and RNA

The *Tip100* transposase coding sequence was amplified from a genomic clone pBK-CMV_4S7_CHSD_Tip100, which was obtained from in vivo excision of λV-Tip100–4S7 (Habu et al. 1998), using primers pCS2-NLS-Tip100-Fw1 and pCS2-Tip100-Rv1 (Table S1). It was subsequently cloned into the *Nco*I and *Xho*I site of the pCS2 + MT vector (Turner and Weintraub 1994), generating the *Tip100* transposase expression vector pCS2-Tip100. mRNA encoding *Tip100* transposase was synthesized from the linearized vector (digested with *Not*I) using mMESSAGE mMACHINE SP6 Kit (Invitrogen) and purified using the Monarch RNA Cleanup Kit (New England Biolabs.).

The original *Tip100* recognition sequences (686 bp at the 5´ end and 1,513 bp at the 3´ end; Figure S1) were amplified form a genomic clone pBK-CMV_4S7_CHSD_Tip100 using primers Tip100-5FW-SpeI and Tip100-5RV-EcoRI for the 5´ end, and Tip100-3FW-SalI and Tip100-3RV-KpnI for the 3´ end (Table S1). The cassette was cloned with a 2.5 kb promoter and a 1.5 kb first intron of the medaka *β-actin* (*actb*) gene (Hamada et al. 1998; Yoshinari et al. 2012), followed by the EGFP gene and an SV40 poly A signal, into the pBluescript vector, generating the ubiquitous expression vector pBS-Tip100-actb4k-EGFP.

For the vector pBS-Tip100-LR100-actb4k-EGFP, which contains truncated recognition sequences (100 bp each), the vector backbone was amplified via inverse PCR using pBS-Tip100-actb4k-EGFP as a template and the primers Tip100-R100-3FW-actb and Tip100-L100-5RV-actb (Table S1). The *actb*-EGFP transgene cassette, amplified using primers actb4kpro-5FW and SV40pA-3RV (Table S1), was cloned into the truncated backbone using In-Fusion Snap Assembly Master Mix (Takara Bio).

For the germline-specific expression vector pBS-Tip100-LR100-olvas-EGFP, the EGFP transgene cassette flanked by a 5.1 kb promoter and a 0.64 kb 3´UTR sequences of medaka *vasa* (*olvas*) (Tanaka et al. 2001) and amplified using the primers olvas-3FW-Tip100 and olvas-5RV-Tip100 (Table S1). The fragment was cloned into the truncated Tip100 backbone vector, which had been amplified using the primers Tip100-R100-3FW-IF and Tip100-L100-5RV-IF (Table S1). The full sequences of the transgenic constructs are available in GitHub repository (https://github.com/satoshi-ansai/Plasmids).

### Microinjection

All plasmid vectors were treated with 0.5% SDS and 0.4 µg/µl proteinase K at 55 °C for 30 min to eliminate residual RNase activity and then purified using the NucleoSpin Gel and PCR Clean-up Kit with Buffer NTB protocol (MACHEREY–NAGEL).

Injection mixtures containing 10 ng/µL of the plasmid vector, with or without 100 ng/µL of *Tip100* mRNA, were introduced into one-cell stage of medaka fertilized eggs as previously described (Kinoshita 2009).

### Inverse PCR

Genomic DNA was extracted from an F_1_ adult fish injected with the pBS-Tip100-LR100-olvas-EGFP vector using the NucleoSpin® Tissue Kit (MACHEREY–NAGEL). Each genomic DNA sample (2 µg) was digested with *Pst*I, purified using the NucleoSpin Gel and PCR Clean-up Kit (MACHEREY–NAGEL), and circularized using Ligation High Ver. 2 (Toyobo). The ligated DNA was used as a PCR template and amplified with primers 5cis-inverse-FW1 and 5cis-inverse-RV1 (Table S1) using KOD FX Neo DNA polymerase (Toyobo). Amplification conditions were as follows: 94 °C for 2 min; 35 cycles of 98 °C for 10 s, 55 °C for 20 s, and 68 °C for 5 min. Subsequently, each PCR product was diluted 400-fold and subjected to a second PCR using primers 5cis-inverse-FW2 and 5cis-inverse-RV2 (Table S1) under the same cycling conditions, except that only 30 cycles were performed. Amplicons were purified via gel extraction using the NucleoSpin Gel and PCR Clean-up Kit and individually sequenced using the primer 5cis-inverse-FW2 at Macrogen Japan. Genomic insertion sites were determined via BLASTN searches against a reference assembly of Japanese medaka in the Ensembl genome browser (ASM223467v1: https://www.ensembl.org/Oryzias_latipes/Info/Index).

To analyze the insertional signature of the *Tip100* transgene cassette, upstream and downstream genomic sequences flanking the cassette were amplified using transgene-specific primers along with 5cis-check-RV and 3cis-check-FW (Table S1). Each amplicon was directly sequenced using the genomic primers.

### Imaging

Injected embryos were observed using a fluorescence stereo microscope M165 FC (Leica Microsystems) with a GFP long-pass filter. Microscopic images were captured with a digital camera FLEXACAM C1 (Leica Microsystems).

### Statistical analyses

All statistical tests were performed using R version 4.4.2.

## Results

**Fig. 1:**
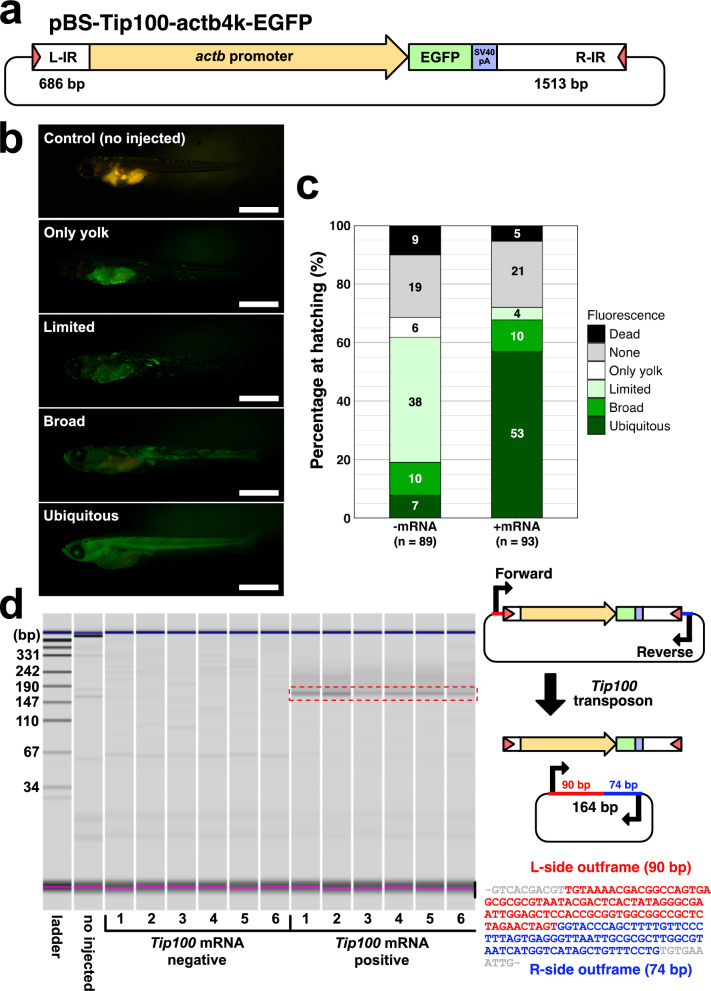
Genomic integration of a transgene by *Tip100* transposon in injected medaka embryos. **a** Schematic representation of the transgenic vector pBS-Tip100-actb4k-EGFP. White boxes with red triangles indicate the recognition sequences with the left (L; 5´) and right (R; 3´) inverted repeats (IRs) of the *Tip100* transposon. The vector contains a 4 kb of medaka *β-actin* (*actb*) promoter sequence followed by an EGFP and an SV40 poly A signal. **b**, **c** Evaluation of the transgene cassette integration efficiency. The pBS-Tip100-actb4k-EGFP vector was injected without (− mRNA) or with (+ mRNA) *Tip100* transposase mRNA. After removing embryos that died before hatching (indicated as “dead”), hatched larvae were classified into five categories based on GFP fluorescence patterns: “control”, “none”, "only yolk", "limited", "broad", or "ubiquitous", defined as follows: no injected control, no fluorescence, fluorescence limited to yolk (absent from the body), fluorescence in less than 50% of the body, fluorescence in more than 50% of the body, or fluorescence in nearly 100% of the body, respectively. **b** Representative images of the four categories of GFP fluorescence expression. Scale bars: 1 mm. **c** Proportion of hatched larvae in each category after injection with ( +) or without ( −) *Tip100* mRNA. The numbers on the bar chart indicate the number of fish in each category. **d** Excision assay to detect *Tip100*-mediated transposition of the transgene cassette. The right panel illustrates the expected PCR product (164 bp) generated following excision of the transgene cassette. In the electrophoresis gel image (left panel), the 164 bp amplicon is highlighted by a red dot square

To investigate whether the *Tip100* transposon can mediate transposition of transgenes in Japanese medaka embryos, we constructed a transgene plasmid, pBS-Tip100-actb4k-EGFP, and an expression vector, pCS2-Tip100, for the transposase. The transgene plasmid contains 686 bp and 1,513 bp of the left (5´) and right (3´) recognition sequences, respectively, which contains terminal inverted repeats essential for transposition of a deleted *Tip100* copy (Ishikawa et al. 2002). It also includes a 4 kb of medaka *beta-actin* (*actb*) promoter driving an EGFP gene as a transgene marker, enabling ubiquitous GFP expression (Hamada et al. 1998), flanked by the recognition sequences (Fig. 1a). To assess the integration efficiency of *Tip100* transposition with conventional (spontaneous) plasmid integration, we injected the transgene plasmid with or without mRNA encoding *Tip100* transposase, transcribed from the transposase expression vector. Injected larvae were categorized into five groups based on the extent of GFP expression: “none”, "only yolk", "limited (< 50% of body)", "broad (> 50%)", or "ubiquitous (nearly 100%)" (Fig. 1b, c). As shown in Fig. 1b, co-injection of the plasmid with *Tip100* mRNA significantly increased the proportion of "broad" and "ubiquitous" larvae (10.75% + 56.99% = 67.74%) compared to those injected without mRNA (11.24% + 7.87% = 19.11%) (Two-sided Fisher’s exact test: *P* = 3.775 × 10^–16^). To confirm that the efficient GFP expression was driven by *Tip100*-mediated transposition, we performed an excision assay to detect transgene cassette excision by PCR amplification (Fig. 1d). A 164 bp PCR product, expected upon excision of the *Tip100* transposon followed by end joining of the DNA breaks, was detected exclusively in embryos injected with *Tip100* mRNA (Fig. 1d). These results indicate that the *Tip100* transposase excises the transgene cassette from the plasmid vector and facilitates its genomic integration in medaka embryos.

**Fig. 2:**
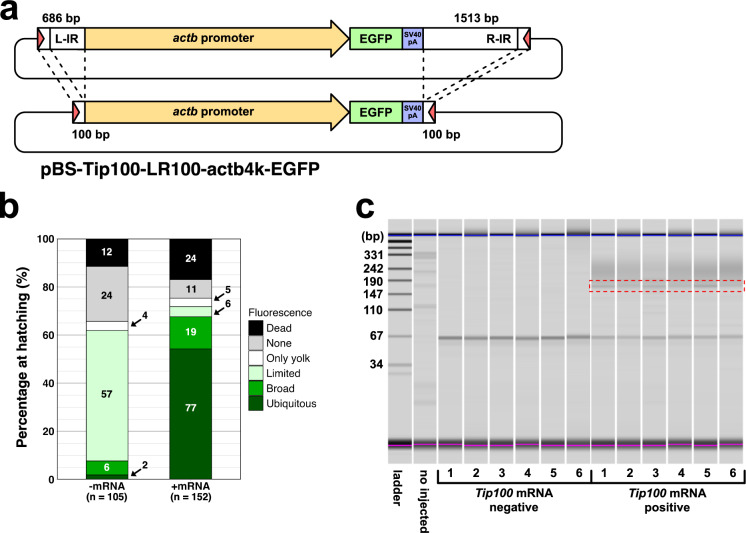
Evaluation of a transgene vector with truncated recognition sequences of the *Tip100* transposon. **a** Schematic representation of the truncated vector pBS-Tip100-LR100-actb4k-EGFP. The left (L; 5´) and right (R; 3´) inverted repeats (IRs) of the *Tip100* transposon are truncated to 100 bp each. The vector contains a 4 kb of medaka *β-actin* (*actb*) promoter sequence followed by an EGFP gene and an SV40 poly A signal. **b** Evaluation of transgene cassette integration efficiency in hatched larvae injected without (− mRNA) or with (+ mRNA) *Tip100* transposase mRNA. After removing embryos that died before hatching (indicated as “dead”), hatched larvae were classified into five categories based on GFP fluorescence patterns: “none”, "only yolk", "limited", "broad", or "ubiquitous" (as defined in Fig. 1b). The numbers on the bar chart indicate the number of fish in each category. **c** Excision assay to detect *Tip100*-mediated transposition of the truncated transgene cassette. The 164 bp PCR product, indicative of transgene cassette excision, is highlighted by a red dotted square

Although *Tip100* shows potential as a novel genetic tool for transgenesis, the total length of the recognition sequences (2.2 kb) limits its practicality for routine molecular cloning. In the *Tol2* transposon system, only 200 bp and 150 bp of the left and the right ends, respectively, are required for the transposition activity (Urasaki et al. 2006). In a native, autonomous copy of *Tip100*, the 3’ recognition site—excluding overlap with the transposase coding region—spans just 118 bp (Ishikawa et al. 2002). Based on this, we constructed a modified transgene plasmid, pBS-Tip100-LR100-act4k-EGFP, containing only 100 bp recognition sequences on each side (Fig. 2a). Injection of this truncated vector with *Tip100* mRNA resulted in a comparable increase in "broad" and "ubiquitous" expression patterns (12.50% + 50.66% = 63.16%) compared to injection without mRNA (5.71% + 1.90% = 7.61%) (Two-sided Fisher’s exact test: *P* < 2.2 × 10^–16^) (Fig. 2b). The 164 bp excision PCR product was again detected only in the presence of *Tip100* mRNA (Fig. 2c), indicating that 100 bp recognition sequences at both ends are sufficient for *Tip100* functional transposition.

**Fig. 3:**
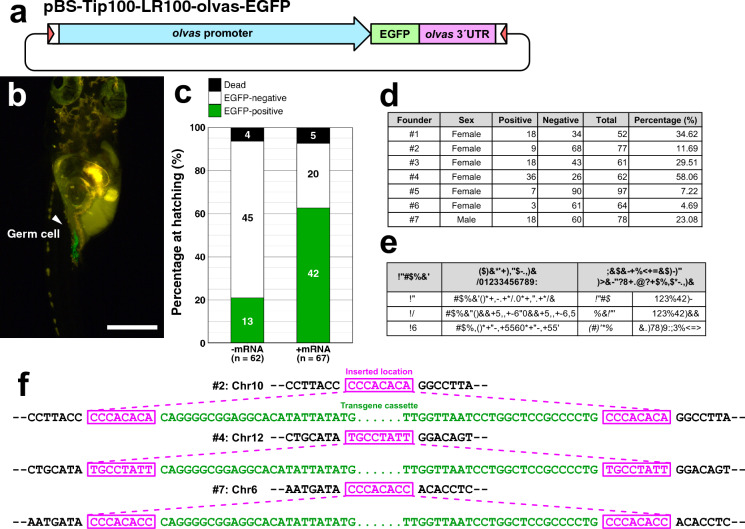
Germline transmission of a transgene cassette integrated by the *Tip100* system. **a** Schematic representation of the pBS-Tip100-LR100-olvas-EGFP vector. Red triangles indicate the left (5´) and right (3´) recognition sequences of the *Tip100* transposon. The vector contains an *EGFP* gene flanked by a 5.1 kb promoter and a 0.64 kb 3´UTR of medaka *vasa* (*olvas*). **b** Representative image of a hatched larva carrying the *olvas:EGFP* cassette, with GFP fluorescence specifically expressed in germ cells. Scale bar: 100 µm. **c** Evaluation of the germline transmission rate of the *Tip100* transgene cassette based on germline-specific GFP fluorescence. Medaka fish injected with the *olvas* vector without (− mRNA) or with (+ mRNA) *Tip100* transposase mRNA were examined. After removing embryos that died before hatching (indicated as “dead”), hatched larvae were classified as EGFP-negative (without fluorescence in germ cells) or EGFP-positive (with fluorescence in germ cells). **d** Germ-line transmission rate in F_1_ offspring obtained by crossing injected fish with wild-type fish. **e** Genomic insertion sites of the transgene cassette determined by inverse PCR in three transgenic strains. **f** Nucleotide sequences of the insertion sites. The 8-bp duplication of the host genome sequence, characteristic of *Tip100* transposon insertions, is highlighted in magenta and enclosed in a square. The sequence of the transgene cassette is highlighted in green letters

We next examined the efficiently of germline transmission of transgenes integrated via the *Tip100* system. To directly visualize germline integration of the transgene through GFP fluorescence, we constructed a plasmid vector, pBS-Tip100-LR100-olvas-EGFP (Fig. 3a), carrying EGFP driven by the promoter and 3´UTR of *Oryzias latipes vasa* (*olvas*), which is specifically expressed in germ cells (Tanaka et al. 2001). As expected, a subset of hatched larvae injected with this transgene plasmid exhibited GFP fluorescence in germ cells (Fig. 3b). Co-injection with the *Tip100* mRNA significantly increased the proportion of larvae with germline GFP signals (mRNA + : 42/68, 62.69%; mRNA−: 13/62, 20.97%) (Two-sided Fisher’s exact test: *P* = 1.278 × 10^–6^) (Fig. 3c). To assess germ-line transmission efficiency to the next generation, seven G_0_ founders with EGFP-positive germ cells were crossed them with wild-type fish. All seven founders produced F_1_ offspring with EGFP-positive germ cells (7/7; 100%), with germline transmission rates ranging from 4.69% (3/64, founder #6) to 58.06% (36/62, founder #4) (Fig. 3d). Finally, we analyzed the genomic integration sites in three strains derived from three different G_0_ founders using inverse PCR followed by Sanger sequencing. Each of the three insertion sites was located on a different chromosomes and showed an 8 bp target site duplication, an insertional signature of the hAT superfamily transposon-mediated integration (Rubin et al. 2001) (Fig. 3e). Together, these results demonstrate that the *Tip100* system enables efficient and stable integration of transgenes into the medaka genome.

## Discussion

In this study, we established a transgenesis method mediated by the *Tip100* system in medaka. To date, two transposon systems, *Ac/Ds* and *Sleeping Beauty*, have been employed for transgenesis in medaka (Grabher et al. 2003; Boon Ng and Gong 2011; Froschauer et al. 2012). While *Sleeping Beauty* has successfully been used to generate enhancer trap strains in medaka, spontaneous integration events independent of the transposon have been observed, resulting in suboptimal integration efficiency (Grabher et al. 2003). A more recent study reported the generation of transgenic strains carrying a single-copy attP landing site for PhiC31-mediated transgenesis using a hyperactive *Sleeping Beauty* transposase (Kirchmaier et al. 2013); however, the integration efficiency was not evaluated. In contrast, the *Ac/Ds* system has been shown to mediate transposon-dependent transgene integration with high efficiency (Boon Ng and Gong 2011; Froschauer et al. 2012; Ishikawa et al. 2018). Nevertheless, the limited accessibility of the *Ac/Ds* plasmids, publicly available only in recent years (Chong-Morrison et al. 2023), has hampered the broader adoption of transposon-mediated transgenesis in the medaka compared to the widespread use of the Tol2 system in zebrafish.

We demonstrated that the *Tip100* system enables efficient integration of transgenes in both somatic (Fig. 1c and 2b) and germline cells (Fig. 3c) of medaka. The observed germline transmission rate is comparable to or exceeds that reported for the *Ac/Ds* system in medaka, where 33–100% of injected fish screened using ubiquitous expression markers are identified as the positive founders (Boon Ng and Gong 2011; Froschauer et al. 2012; Ishikawa et al. 2018). It is also on par with the *Tol2* system in zebrafish, which yields 50–70% positive founders (Kawakami et al. 2004; Urasaki et al. 2006). Furthermore, while highly effective transgenesis with transposase mRNA is often associated with increased mortality in injected embryos (Ishikawa et al. 2018), *Tip100* transposase mRNA did not elevate mortality rates (5–16% of injected fish; Fig. 1c, 2b, and 3c). Notably, plasmids for the *Tip100* system are publicly available through the National BioResource Project (NBRP) Morning Glory (https://shigen.nig.ac.jp/asagao/), promoting accessibility. The *Tip100* system also offers a practical advantage in vector construction: it requires only total 200 bp of recognition sequences, the shortest among transposon systems used in fish. In comparison, *Ac/Ds* requires 609 bp (Emelyanov et al. 2006), *Sleeping Beauty* 455 bp (Zayed et al. 2004), and *Tol2* 350 bp (Urasaki et al. 2006). This reduced sequence length facilitates the construction of more compact plasmids.

Taken together, our findings support the utility of *Tip100* as a genetic tool for efficient and stable transgenesis in medaka, and potentially other organisms. However, several additional features of the system need to be clarified for broader application. First, the cargo size capacity is a critical property of transposon-based tools. In this study, 4.7- and 6.7-kb transgene cassettes were efficiently integrated into the medaka genome, indicating that *Tip100* can accommodate inserts suitable for conventional plasmid-based transgenesis (< 10 kb). Further studies are required to determine whether *Tip100* can also mediate the insertion of larger genomic clones, such as bacterial artificial chromosome (BAC) and Fosmid clones, as reported for other transposon systems (Suster et al. 2009; Rostovskaya et al. 2012). Second, insertion site preference can influence both the stability of the transgene expression and the suitability of the system for applications such as gene and enhancer trapping. Among the three alleles for which insertion sites were identified, two were located in introns on protein coding genes, while one was in an intergenic region (Fig. 3e). Although the sample size is too limited to draw firm conclusions, *Tip100* may preferentially insert into gene-rich or otherwise open chromatin regions. Finally, it remains important to test whether *Tip100*-mediated transgenesis is effective in other animal models. Because transposon systems have also been applied to genetic manipulation in cultured cells, future studies should examine *Tip100* activity in such systems to assess its broader potential as a genetic tool.

## Supplementary Information

Below is the link to the electronic supplementary material.

Supplementary file1 (PDF 69 KB)

Supplementary file2 (XLSX 10 KB)

## Data Availability

Full sequences of the plasmid generated in this study are available in a GitHub repository (https://github.com/satoshi-ansai/Plasmids/tree/main/Tip100).

## References

[CR1] Asakawa K, Suster ML, Mizusawa K et al (2008) Genetic dissection of neural circuits by Tol2 transposon-mediated Gal4 gene and enhancer trapping in zebrafish. Proc Natl Acad Sci U S A 105:1255–1260 18202183 10.1073/pnas.0704963105 PMC2234125

[CR2] Boon Ng GH, Gong Z (2011) Maize Ac/Ds transposon system leads to highly efficient germline transmission of transgenes in medaka (*Oryzias latipes*). Biochimie 93:1858–1864 21777650 10.1016/j.biochi.2011.07.006

[CR3] Chong-Morrison V, Mayes S, Simões FC et al (2023) Ac/Ds transposition for CRISPR/dCas9-SID4x epigenome modulation in zebrafish. Biol Open. 10.1242/bio.059995 37367831 10.1242/bio.059995 PMC10320716

[CR4] Emelyanov A, Gao Y, Naqvi NI, Parinov S (2006) Trans-kingdom transposition of the maize dissociation element. Genetics 174:1095–1104 16951067 10.1534/genetics.106.061184 PMC1667081

[CR5] Erickson PA, Ellis NA, Miller CT (2016) Microinjection for transgenesis and genome editing in threespine sticklebacks. J vis Exp. 10.3791/54055 27214565 10.3791/54055 PMC4942152

[CR6] Froschauer A, Sprott D, Gerwien F et al (2012) Effective generation of transgenic reporter and gene trap lines of the medaka (*Oryzias latipes*) using the Ac/Ds transposon system. Transgenic Res 21:149–162 21533666 10.1007/s11248-011-9514-x

[CR7] Fujimura K, Kocher TD (2011) Tol2-mediated transgenesis in tilapia (*Oreochromis niloticus*). Aquaculture 319:342–346 21938082 10.1016/j.aquaculture.2011.07.021 PMC3175368

[CR8] Grabher C, Henrich T, Sasado T et al (2003) Transposon-mediated enhancer trapping in medaka. Gene 322:57–66 14644497 10.1016/j.gene.2003.09.009

[CR9] Habu Y, Hisatomi Y, Iida S (1998) Molecular characterization of the mutable flaked allele for flower variegation in the common morning glory. Plant J 16:371–376 9881157 10.1046/j.1365-313x.1998.00308.x

[CR10] Hamada K, Tamaki K, Sasado T et al (1998) Usefulness of the medaka beta-actin promoter investigated using a mutant GFP reporter gene in transgenic medaka (Oryzias latipes). Mol Mar Biol Biotechnol 7:173–180 9701611

[CR11] Higaki S, Nishie T, Todo T et al (2021) Germ cell-specific expression of Venus by Tol2-mediated transgenesis in endangered endemic cyprinid Honmoroko (Gnathopogon caerulescens). J Fish Biol 99:1341–1347 34189725 10.1111/jfb.14840

[CR12] Iida A, Takamatsu N, Hori H et al (2005) Reversion mutation of Ib oculocutaneous albinism to wild-type pigmentation in medaka fish. Pigment Cell Res 18:382–384 16162178 10.1111/j.1600-0749.2005.00247.x

[CR13] Ishikawa N, Johzuka-Hisatomi Y, Sugita K et al (2002) The transposon Tip100 from the common morning glory is an autonomous element that can transpose in tobacco plants. Mol Genet Genomics 266:732–739 11810246 10.1007/s00438-001-0603-z

[CR14] Ishikawa T, Ansai S, Kinoshita M, Mori K (2018) A collection of transgenic Medaka strains for efficient site-directed transgenesis mediated by phiC31 integrase. G3 Genes|genomes|genetics 8:2585–2593 29848622 10.1534/g3.118.200130 PMC6071608

[CR15] Ivics Z, Li MA, Mátés L et al (2009) Transposon-mediated genome manipulation in vertebrates. Nat Methods 6:415–422 19478801 10.1038/nmeth.1332 PMC2867038

[CR16] Juntti SA, Hu CK, Fernald RD (2013) Tol2-mediated generation of a transgenic haplochromine cichlid, *Astatotilapia burtoni*. PLoS ONE 8:e77647 24204902 10.1371/journal.pone.0077647 PMC3808393

[CR17] Kawakami K (2005) Transposon tools and methods in zebrafish. Dev Dyn 234:244–254 16110506 10.1002/dvdy.20516

[CR18] Kawakami K, Takeda H, Kawakami N et al (2004) A transposon-mediated gene trap approach identifies developmentally regulated genes in zebrafish. Dev Cell 7:133–144 15239961 10.1016/j.devcel.2004.06.005

[CR19] Kinoshita M (2009) Medaka: biology, management, and experimental protocols. Wiley-Blackwell, Ames, Iowa

[CR20] Kirchmaier S, Höckendorf B, Möller EK et al (2013) Efficient site-specific transgenesis and enhancer activity tests in medaka using PhiC31 integrase. Development 140:4287–4295 24048591 10.1242/dev.096081 PMC3809364

[CR21] Kirchmaier S, Naruse K, Wittbrodt J, Loosli F (2015) The genomic and genetic toolbox of the teleost medaka (*Oryzias latipes*). Genetics 199:905–918 25855651 10.1534/genetics.114.173849 PMC4391551

[CR22] Koga A, Suzuki M, Inagaki H et al (1996) Transposable element in fish. Nature 383:30–30 8779712 10.1038/383030a0

[CR23] Koga A, Iida A, Hori H et al (2006) Vertebrate DNA transposon as a natural mutator: the medaka fish Tol2 element contributes to genetic variation without recognizable traces. Mol Biol Evol 23:1414–1419 16672286 10.1093/molbev/msl003

[CR24] Miyamoto K, Abe G, Kawakami K et al (2024) The dwarf neon rainbowfish *Melanotaenia praecox*, a small spiny-rayed fish with potential as a new Acanthomorpha model fish: II. Establishment of a microinjection procedure for genetic engineering. Dev Dyn 253:815–828 38314924 10.1002/dvdy.698 PMC11656680

[CR25] Rostovskaya M, Fu J, Obst M et al (2012) Transposon-mediated BAC transgenesis in human ES cells. Nucleic Acids Res 40:e150 22753106 10.1093/nar/gks643 PMC3479164

[CR26] Rubin E, Lithwick G, Levy AA (2001) Structure and evolution of the hAT transposon superfamily. Genetics 158:949–957 11454746 10.1093/genetics/158.3.949 PMC1461711

[CR27] Shen D, Song C, Miskey C et al (2021) A native, highly active Tc1/mariner transposon from zebrafish (ZB) offers an efficient genetic manipulation tool for vertebrates. Nucleic Acids Res 49:2126–2140 33638993 10.1093/nar/gkab045 PMC7913693

[CR28] Suster ML, Sumiyama K, Kawakami K (2009) Transposon-mediated BAC transgenesis in zebrafish and mice. BMC Genomics 10:477 19832998 10.1186/1471-2164-10-477 PMC2768751

[CR29] Takeda H, Shimada A (2010) The art of medaka genetics and genomics: What makes them so unique? Annu Rev Genet 44:217–241 20731603 10.1146/annurev-genet-051710-151001

[CR30] Tanaka M, Kinoshita M, Kobayashi D, Nagahama Y (2001) Establishment of medaka (*Oryzias latipes*) transgenic lines with the expression of green fluorescent protein fluorescence exclusively in germ cells: a useful model to monitor germ cells in a live vertebrate. Proc Natl Acad Sci U S A 98:2544–2549 11226275 10.1073/pnas.041315498 PMC30174

[CR31] Turner DL, Weintraub H (1994) Expression of *achaete-scute* homolog 3 in *Xenopus* embryos converts ectodermal cells to a neural fate. Genes Dev 8:1434–1447 7926743 10.1101/gad.8.12.1434

[CR32] Urasaki A, Morvan G, Kawakami K (2006) Functional dissection of the Tol2 transposable element identified the minimal cis-sequence and a highly repetitive sequence in the subterminal region essential for transposition. Genetics 174:639–649 16959904 10.1534/genetics.106.060244 PMC1602067

[CR33] Valenzano DR, Sharp S, Brunet A (2011) Transposon-mediated transgenesis in the short-lived African killifish *Nothobranchius furzeri*, a vertebrate model for aging. G3 (Bethesda) 1:531–538 22384364 10.1534/g3.111.001271 PMC3276177

[CR34] Wang S, Gao B, Miskey C et al (2023) Passer, a highly active transposon from a fish genome, as a potential new robust genetic manipulation tool. Nucleic Acids Res 51:1843–1858 36688327 10.1093/nar/gkad005 PMC9976928

[CR35] Yoshinari N, Ando K, Kudo A et al (2012) Colored medaka and zebrafish: transgenics with ubiquitous and strong transgene expression driven by the medaka β-actin promoter. Dev Growth Differ 54:818–828 23157381 10.1111/dgd.12013

[CR36] Zayed H, Izsvák Z, Walisko O, Ivics Z (2004) Development of hyperactive sleeping beauty transposon vectors by mutational analysis. Mol Ther 9:292–304 14759813 10.1016/j.ymthe.2003.11.024

