# Supplementary material for: Highly efficient transgenesis mediated by Tip100 transposon system in medaka
Source: Transgenic Res. 2025 Oct 9;34(1):46. doi: 10.1007/s11248-025-00466-5 (PMC12511174; doi:10.1007/s11248-025-00466-5)
Supplement: Supplementary file 1 — Supplementary file1 (PDF 69 KB) [file 11248_2025_466_MOESM1_ESM.pdf]

Supplementary Figure S1. A full sequence of 3.8 kb functional copy of *Tip100* transposon.

```
Thu Sep 18, 2025 17:49 JST
Tip100_functional_copy.gb
                10        20        30        40        50        60        70        80        90       100       110       120       130       140       150
                 *    *    *    *    *    *    *    *    *    *    *    *    *    *    *    *    *    *    *    *    *    *    *    *    *    *    *    *    *    *
     1 CAGGGGCGGAGGCACATATTATATGCAGGGGGCCTTGGCCCCCCCCCCCAACAAAAAAAATTTTAATGCATGCAATTAGTATAGCCTATTAGGCAATAGGGGCCTTGGCGGCTTGGCCCCCCCCCAAAAAAAAAAAAAAAAAAAAATATTA 150
       >>>>>>>>>>>
       terminal inverted repeat
       >>>>>>>>>>>>>>>>>>>>>>>>>>>>>>>>>>>>>>>>>>>>>>>>>>>>>>>>>>>>>>>>>>>>>>>>>>>>>>>>>>>>>>>>>>>>>>>>>>>>>>>>>>>>>>>>>>>>>>>>>>>>>>>>>>>>>>>>>>>>>>>>>>>>>>>>
       Left recognition sequence (686 bp)
       >>>>>>>>>>>>>>>>>>>>>>>>>>>>>>>>>>>>>>>>>>>>>>>>>>>>>>>>>>>>>>>>>>>>>>>>>>>>>>>>>>>>>>>>>>>>>
       Left recognition sequence (100 bp)

               160       170       180       190       200       210       220       230       240       250       260       270       280       290       300
                 *    *    *    *    *    *    *    *    *    *    *    *    *    *    *    *    *    *    *    *    *    *    *    *    *    *    *    *    *    *
   151 GTAGTGTAATTAATTGTGGCCCTTCACCAATCAGTAATCATCCAATATTTTCCCTCCCCAAACAAAGCTTCCTTTGTAAGCTTTCACTTAACCCAATTTCAAACCAAACCTGCAACCCGCATATACGCAAATCAAATTTGGAAGAAGATA 300
       >>>>>>>>>>>>>>>>>>>>>>>>>>>>>>>>>>>>>>>>>>>>>>>>>>>>>>>>>>>>>>>>>>>>>>>>>>>>>>>>>>>>>>>>>>>>>>>>>>>>>>>>>>>>>>>>>>>>>>>>>>>>>>>>>>>>>>>>>>>>>>>>>>>>>>>>
       Left recognition sequence (686 bp)

               310       320       330       340       350       360       370       380       390       400       410       420       430       440       450
                 *    *    *    *    *    *    *    *    *    *    *    *    *    *    *    *    *    *    *    *    *    *    *    *    *    *    *    *    *    *
   301 ATAAATACACTCAAATCACGAATCAAAGCAATACACACAAGCCATTTCTCTTCCATTTTCAAGCAGCAAATGTATAGGTAATTTTACATTTCTTTTTTATTATTTAAATTTTTGAATTTTTTTTTTATGAATTATTCTTTATTGGAGATA 450
       >>>>>>>>>>>>>>>>>>>>>>>>>>>>>>>>>>>>>>>>>>>>>>>>>>>>>>>>>>>>>>>>>>>>>>>>>>>>>>>>>>>>>>>>>>>>>>>>>>>>>>>>>>>>>>>>>>>>>>>>>>>>>>>>>>>>>>>>>>>>>>>>>>>>>>>>
       Left recognition sequence (686 bp)

               460       470       480       490       500       510       520       530       540       550       560       570       580       590       600
                 *    *    *    *    *    *    *    *    *    *    *    *    *    *    *    *    *    *    *    *    *    *    *    *    *    *    *    *    *    *
   451 AATTCATCATTCTTCACTACAATAAAAAGTCTAGATATTGTAGTTCAAGGGTAGCTAAATGCCTAAATTCTCTCTTTCGTTTCTTCAGTTGACGACGGCAACCGTGGTGAACTCGGGCTACTGGCTACGAGTAGTGCCGACTGGAAGCTG 600
       >>>>>>>>>>>>>>>>>>>>>>>>>>>>>>>>>>>>>>>>>>>>>>>>>>>>>>>>>>>>>>>>>>>>>>>>>>>>>>>>>>>>>>>>>>>>>>>>>>>>>>>>>>>>>>>>>>>>>>>>>>>>>>>>>>>>>>>>>>>>>>>>>>>>>>>>
       Left recognition sequence (686 bp)

               610       620       630       640       650       660       670       680       690       700       710       720       730       740       750
                 *    *    *    *    *    *    *    *    *    *    *    *    *    *    *    *    *    *    *    *    *    *    *    *    *    *    *    *    *    *
                                                                                         687 SpeI
                                                                                         |
   601 GATCCTGTTTGGAGTGTCGGCGTTCTCCTTTTGTTGGTGTATGTTTATACTTTTAAGAGTTCTATAAGATTTGCTAGAATTTAAATACTAGTGCATAATGAAATGGGTCATTTGATGAGTTTTGAAAATGATAGTATTAGTTGAGGATTA 750
       >>>>>>>>>>>>>>>>>>>>>>>>>>>>>>>>>>>>>>>>>>>>>>>>>>>>>>>>>>>>>>>>>>>>>>>>>>>>>>>>
       Left recognition sequence (686 bp)

               760       770       780       790       800       810       820       830       840       850       860       870       880       890       900
                 *    *    *    *    *    *    *    *    *    *    *    *    *    *    *    *    *    *    *    *    *    *    *    *    *    *    *    *    *    *
   751 TATGCCATTGTTGTGGATTTGAGATGATGTTATAATTACAGAAAGGGTCAGTGTTATATATGATGATATTAGCTTGGAGAAAGAGATTAGAAGCTAGCATATTCAATTCATGCAGACAAAAAAATTAGATAATTAGAATGCCAAATCAACT 900

               910       920       930       940       950       960       970       980       990      1000      1010      1020      1030      1040      1050
                 *    *    *    *    *    *    *    *    *    *    *    *    *    *    *    *    *    *    *    *    *    *    *    *    *    *    *    *    *    *
   901 TAAATTAATATTTAGTTAAGAAGAAGTTAGAACCATTGATGTTACTACTCTATCTTTTCATAAACAACATAATATATAATTTATATAGTATATATAGTTTATGCATGAGGAGTAATTAGGTGATTTAATGAGTTTTGAAAATGGCAGTAC 1050

              1060      1070      1080      1090      1100      1110      1120      1130      1140      1150      1160      1170      1180      1190      1200
                 *    *    *    *    *    *    *    *    *    *    *    *    *    *    *    *    *    *    *    *    *    *    *    *    *    *    *    *    *    *
  1051 ACAGTATTAATTAGGATTTATTTGTCTTTATTAATTTGGTGATTTGATGTGTTTATTTACTCCTGTATTATATATAGTTAATATAATTGTGTTTAATTAGTATATTAATTGACAATACACAATATTGATTTTGATGAGTATTAATTGTATTA 1200

              1210      1220      1230      1240      1250      1260      1270      1280      1290      1300      1310      1320      1330      1340      1350
                 *    *    *    *    *    *    *    *    *    *    *    *    *    *    *    *    *    *    *    *    *    *    *    *    *    *    *    *    *    *
  1201 ATTGTATTTATTATATAATTAGTATATTAATTGGCACTTGGCAGTACACAATATCTTTGTTTGTTTATTTTTTTTTCCTCAATTAATTTGTCAAAATATTAACAGTGTGAACATGAGAAGATTGAGTTGAAATGAAAAAGACACAAAAAATAG 1350
                                                                                                                                   M  K  K  T  Q  K  I  D
                                                                                                                                   1
                                                                                                                                   >>>>>>>>>>>>>>>>>>>>>>
                                                                                                                                   Tip100 transposase

              1360      1370      1380      1390      1400      1410      1420      1430      1440      1450      1460      1470      1480      1490      1500
                 *    *    *    *    *    *    *    *    *    *    *    *    *    *    *    *    *    *    *    *    *    *    *    *    *    *    *    *    *    *
  1351 ATCTTTTCTTTAAAAGAAAGAATTCTGAAGATTCAACATCTCAAGTAAATCCATTTGCAAGTATTGAACCATTGAATTCAGAAAGTCGTCCTGAAAAAGCTCAAAGAGTTGAAATCAATGAAAAGTTTGATATTCAAGCATTAGAACGCG 1500
         L  F  F  K  R  K  N  S  E  D  S  T  S  Q  V  N  P  F  A  S  I  E  P  L  N  S  E  S  R  P  E  K  A  Q  R  V  E  I  N  E  K  F  D  I  Q  A  L  E  R  D
       9
       >>>>>>>>>>>>>>>>>>>>>>>>>>>>>>>>>>>>>>>>>>>>>>>>>>>>>>>>>>>>>>>>>>>>>>>>>>>>>>>>>>>>>>>>>>>>>>>>>>>>>>>>>>>>>>>>>>>>>>>>>>>>>>>>>>>>>>>>>>>>>>>>>>>>>>>>
       Tip100 transposase

              1510      1520      1530      1540      1550      1560      1570      1580      1590      1600      1610      1620      1630      1640      1650
                 *    *    *    *    *    *    *    *    *    *    *    *    *    *    *    *    *    *    *    *    *    *    *    *    *    *    *    *    *    *
  1501 ATCCAGGATTGCGACTACCAATATGGAAGTGCCCCATTGAGAAGCGTGATGAAGTTAGGAGAGCTTATATCAAGGCTGGCCCATATCAATGTTTGCTTTCAAAGTATCCAAAATCGGGAGAAAAGCATCCTCGTAGTTTTCAGGCATCTT 1650
         P  G  L  R  L  P  I  W  K  C  P  I  E  K  R  D  E  V  R  R  A  Y  I  K  A  G  P  Y  Q  C  L  L  S  K  Y  P  K  S  G  E  K  H  P  R  S  F  Q  A  S  W
       59
       >>>>>>>>>>>>>>>>>>>>>>>>>>>>>>>>>>>>>>>>>>>>>>>>>>>>>>>>>>>>>>>>>>>>>>>>>>>>>>>>>>>>>>>>>>>>>>>>>>>>>>>>>>>>>>>>>>>>>>>>>>>>>>>>>>>>>>>>>>>>>>>>>>>>>>>>
       Tip100 transposase
```

Supplementary Figure S1. (Continued)

```
1651 GGTTTAAGTTATTCCCTTCTTGGTTAGAATATTCTCCTAGTAAAGATGCAGCATTTTGTCTACCATGTTATTTGTTTCATACTCAAGATGGGCCTTCCGGATTGGATGCTTTTACTATCAATGGGTTTAGATCATGGAAGAAAGTTAGAG 1800
       F  K  L  F  P  S  W  L  E  Y  S  P  S  K  D  A  A  F  C  L  P  C  Y  L  F  H  T  Q  D  G  P  S  G  L  D  A  F  T  I  N  G  F  R  S  W  K  K  V  R  D
     109
     >>>>>>>>>>>>>>>>>>>>>>>>>>>>>>>>>>>>>>>>>>>>>>>>>>>>>>>>>>>>>>>>>>>>>>>>>>>>>>>>>>>>>>>>>>>>>>>>>>>>>>>>>>>>>>>>>>>>>>>>>>>>>>>>>>>>>>>>>>>>>>>>>>>>>>
     Tip100 transposase

1801 ATGGAAAAAATTGCTCATTTTTAGCACATATTGGGAAAGATCTCACCTCCCCTCACCGAAATGCCGAGAAAGCTTGTGCGGATCTCATGAACCAACAATCACACATTGCACAACATATTGAGAAATTCACATCTCAAGAAATAGCAGAGA 1950
       G  K  N  C  S  F  L  A  H  I  G  K  D  L  T  S  P  H  R  N  A  E  K  A  C  A  D  L  M  N  Q  Q  S  H  I  A  Q  H  I  E  K  F  T  S  Q  E  I  A  E  N
     159
     >>>>>>>>>>>>>>>>>>>>>>>>>>>>>>>>>>>>>>>>>>>>>>>>>>>>>>>>>>>>>>>>>>>>>>>>>>>>>>>>>>>>>>>>>>>>>>>>>>>>>>>>>>>>>>>>>>>>>>>>>>>>>>>>>>>>>>>>>>>>>>>>>>>>>>
     Tip100 transposase

1951 ATAGACTTCGCTTGAGAACTTCAATTGAGGCAACAAGATGGCTTGCATTTCAAGGATGTTCTTTTAGAGGTCATGATGAGTCAATAACATCAACGAACCGTGGAAACTTTTTAGAGCTATTGAGTTTCATTGCCTCTTTTAATGATGAAG 2100
       R  L  R  L  R  T  S  I  E  A  T  R  W  L  A  F  Q  G  C  S  F  R  G  H  D  E  S  I  T  S  T  N  R  G  N  F  L  E  L  L  S  F  I  A  S  F  N  D  E  V
     209
     >>>>>>>>>>>>>>>>>>>>>>>>>>>>>>>>>>>>>>>>>>>>>>>>>>>>>>>>>>>>>>>>>>>>>>>>>>>>>>>>>>>>>>>>>>>>>>>>>>>>>>>>>>>>>>>>>>>>>>>>>>>>>>>>>>>>>>>>>>>>>>>>>>>>>>
     Tip100 transposase

2101 TAACAAAAGTTCTTGATAAAGCTCCACGAAATGCATCATATACATCACCAACAATACAAAAGCAAATTTTGCAAGTGTTGGCAACTAAAGTAAAAAGTGCAATCCGAGAAGAAATTGGTGATGCAAAATTTTGTATCATTGTTGATGAAG 2250
       T  K  V  L  D  K  A  P  R  N  A  S  Y  T  S  P  T  I  Q  K  Q  I  L  Q  V  L  A  T  K  V  K  S  A  I  R  E  E  I  G  D  A  K  F  C  I  I  V  D  E  A
     259
     >>>>>>>>>>>>>>>>>>>>>>>>>>>>>>>>>>>>>>>>>>>>>>>>>>>>>>>>>>>>>>>>>>>>>>>>>>>>>>>>>>>>>>>>>>>>>>>>>>>>>>>>>>>>>>>>>>>>>>>>>>>>>>>>>>>>>>>>>>>>>>>>>>>>>>
     Tip100 transposase

                                                                                                              2355 SpeI
                                                                                                              |
2251 CTCGAGATGAATCAAAGAAGGAACAAATGTCAATTGTCTTGAGATTTGTTGATAGAGATGGCTTTATACAAGAGCGTTTCTTTGGAGTTGTTCATGTTAAGGACACTAGTGCATCAACATTGAAAGAGGGCATCTTTTCTATCTTATCTC 2400
       R  D  E  S  K  K  E  Q  M  S  I  V  L  R  F  V  D  R  D  G  F  I  Q  E  R  F  F  G  V  V  H  V  K  D  T  S  A  S  T  L  K  E  G  I  F  S  I  L  S  R
     309
     >>>>>>>>>>>>>>>>>>>>>>>>>>>>>>>>>>>>>>>>>>>>>>>>>>>>>>>>>>>>>>>>>>>>>>>>>>>>>>>>>>>>>>>>>>>>>>>>>>>>>>>>>>>>>>>>>>>>>>>>>>>>>>>>>>>>>>>>>>>>>>>>>>>>>>
     Tip100 transposase
                                                                                                              <<<<<<<<<<<<<<<<<<<<<<<<<<<<<<<<<<<<<<<<
                                                                                                              Right recognition sequence (1513 bp)

2401 GTCATAATCTTGATGTTCAAAATATCCGGGGACAAGGTTATGATGGTGCAAGTAACATGCGTGGAGAATGGAATGGATTGAAAGCTTTAATTTTGGATGAATGTCCTTATGCTTATTATGTTCATTGTTTTGCACATCGATTGCAATTGG 2550
       H  N  L  D  V  Q  N  I  R  G  Q  G  Y  D  G  A  S  N  M  R  G  E  W  N  G  L  K  A  L  I  L  D  E  C  P  Y  A  Y  Y  V  H  C  F  A  H  R  L  Q  L  A
     359
     >>>>>>>>>>>>>>>>>>>>>>>>>>>>>>>>>>>>>>>>>>>>>>>>>>>>>>>>>>>>>>>>>>>>>>>>>>>>>>>>>>>>>>>>>>>>>>>>>>>>>>>>>>>>>>>>>>>>>>>>>>>>>>>>>>>>>>>>>>>>>>>>>>>>>>
     Tip100 transposase
     <<<<<<<<<<<<<<<<<<<<<<<<<<<<<<<<<<<<<<<<<<<<<<<<<<<<<<<<<<<<<<<<<<<<<<<<<<<<<<<<<<<<<<<<<<<<<<<<<<<<<<<<<<<<<<<<<<<<<<<<<<<<<<<<<<<<<<<<<<<<<<<<<<<<<<
     Right recognition sequence (1513 bp)

2551 CATTAGTAGCTTCATCAAAGGAAGTCATCCCTGTCCATCAGTTTTTCACTAAGTTAAACTCCATTATTAATGTTGTTGGGGCTTCATGTAAGCGCAATGACCAATTAAAAGCTGCTCATGCCTCAAATATTTCTCATTTGCTGAGTATTG 2700
       L  V  A  S  S  K  E  V  I  P  V  H  Q  F  F  T  K  L  N  S  I  I  N  V  V  G  A  S  C  K  R  N  D  Q  L  K  A  A  H  A  S  N  I  S  H  L  L  S  I  D
     409
     >>>>>>>>>>>>>>>>>>>>>>>>>>>>>>>>>>>>>>>>>>>>>>>>>>>>>>>>>>>>>>>>>>>>>>>>>>>>>>>>>>>>>>>>>>>>>>>>>>>>>>>>>>>>>>>>>>>>>>>>>>>>>>>>>>>>>>>>>>>>>>>>>>>>>>
     Tip100 transposase
     <<<<<<<<<<<<<<<<<<<<<<<<<<<<<<<<<<<<<<<<<<<<<<<<<<<<<<<<<<<<<<<<<<<<<<<<<<<<<<<<<<<<<<<<<<<<<<<<<<<<<<<<<<<<<<<<<<<<<<<<<<<<<<<<<<<<<<<<<<<<<<<<<<<<<<
     Right recognition sequence (1513 bp)

2701 ATGAGCTAGAAAGTGGGAGGGGCCTTAACCAAATTGGTTCTTTACAAAGGCCAGGTGATACTCGCTGGAGTTCTCATTTGAAATCTATATCAAGTTTGATGAGAATGTTTAGTGCAACATGTGAAGTTTTACTTAATATTATTGAAGATG 2850
       E  L  E  S  G  R  G  L  N  Q  I  G  S  L  Q  R  P  G  D  T  R  W  S  S  H  L  K  S  I  S  S  L  M  R  M  F  S  A  T  C  E  V  L  L  N  I  I  E  D  G
     459
     >>>>>>>>>>>>>>>>>>>>>>>>>>>>>>>>>>>>>>>>>>>>>>>>>>>>>>>>>>>>>>>>>>>>>>>>>>>>>>>>>>>>>>>>>>>>>>>>>>>>>>>>>>>>>>>>>>>>>>>>>>>>>>>>>>>>>>>>>>>>>>>>>>>>>>
     Tip100 transposase
     <<<<<<<<<<<<<<<<<<<<<<<<<<<<<<<<<<<<<<<<<<<<<<<<<<<<<<<<<<<<<<<<<<<<<<<<<<<<<<<<<<<<<<<<<<<<<<<<<<<<<<<<<<<<<<<<<<<<<<<<<<<<<<<<<<<<<<<<<<<<<<<<<<<<<<
     Right recognition sequence (1513 bp)

2851 GAACTACACATGCTCACCGTGGAGATGCCGATGCAGCTTATGAGGTACTCACTTCTTTTGAATTTGTATTCATTATGCATCTCATGAAAAAAGTTTTGGAGATTTCTAATATGCTTTGCCAAGCTTTGCAACTTCAATCTCAAGATATAT 3000
       T  T  H  A  H  R  G  D  A  D  A  A  Y  E  V  L  T  S  F  E  F  V  F  I  M  H  L  M  K  K  V  L  E  I  S  N  M  L  C  Q  A  L  Q  L  Q  S  Q  D  I  L
     509
     >>>>>>>>>>>>>>>>>>>>>>>>>>>>>>>>>>>>>>>>>>>>>>>>>>>>>>>>>>>>>>>>>>>>>>>>>>>>>>>>>>>>>>>>>>>>>>>>>>>>>>>>>>>>>>>>>>>>>>>>>>>>>>>>>>>>>>>>>>>>>>>>>>>>>>
```

Supplementary Figure S1. (Continued)

```
Tip100 transposase
Right recognition sequence (1513 bp)

        3010      3020      3030      3040      3050      3060      3070      3080      3090      3100      3110      3120      3130      3140      3150
3001 TGAATGCAATGCATCTTGTATCATCTACTAAATTGCTTATTCAAACTTTAAGAGATAGTGGATGGGATGAATTAGTTGCAAGTGTGAAGTCTTTTTGTGAAACTGTTAATATAACTGTGCCGGATTTTGATGCTCAATATATTGCAAGAA 3150
      N  A  M  H  L  V  S  S  T  K  L  L  I  Q  T  L  R  D  S  G  W  D  E  L  V  A  S  V  K  S  F  C  E  T  V  N  I  T  V  P  D  F  D  A  Q  Y  I  A  R  R
     559
     Tip100 transposase
     Right recognition sequence (1513 bp)

        3160      3170      3180      3190      3200      3210      3220      3230      3240      3250      3260      3270      3280      3290      3300
3151 GAGGAAGGGCTAGACATCAACAAGATGAATTAACAATTGGGCATCATTACAAAGTTGATATTTTTTATGCGGTGATTGATTCTCAGTTGCAAGAGTTGAGCAATAGGTTTGATCATAAAGCAATGGAGTTAATTGTTCTCAGTTCATCGT 3300
      G  R  A  R  H  Q  Q  D  E  L  T  I  G  H  H  Y  K  V  D  I  F  Y  A  V  I  D  S  Q  L  Q  E  L  S  N  R  F  D  H  K  A  M  E  L  I  V  L  S  S  S  L
     609
     Tip100 transposase
     Right recognition sequence (1513 bp)

        3310      3320      3330      3340      3350      3360      3370      3380      3390      3400      3410      3420      3430      3440      3450
3301 TAGATCCAAAAGAGATGCGTATATCATTCAGAATTGATGATGTTTGCAAGTTGGTAGAGAAGTTTTACCCACAAGACTTTGAAGATTATGAGACTTTGCAATTGAGAGTGCAACTTGAACATTTTGAACATGTGCAACAACTTCCTGATT 3450
      D  P  K  E  M  R  I  S  F  R  I  D  D  V  C  K  L  V  E  K  F  Y  P  Q  D  F  E  D  Y  E  T  L  Q  L  R  V  Q  L  E  H  F  E  H  V  Q  Q  L  P  D  F
     659
     Tip100 transposase
     Right recognition sequence (1513 bp)

        3460      3470      3480      3490      3500      3510      3520      3530      3540      3550      3560      3570      3580      3590      3600
3451 TTAGAACACTAGAAAGTATTTCTGATCTATGCCGATGGTTGGTAAAAACTAGAAAATCGAACATTTATCCTCTTGTGTTCAGAGTAGTAACTCTCGTTCTCACACTTCCAGTATCTACAGCTACTACAGAACGATCTTTTTCTGCTATGA 3600
      R  T  L  E  S  I  S  D  L  C  R  W  L  V  K  T  R  K  S  N  I  Y  P  L  V  F  R  V  V  T  L  V  L  T  L  P  V  S  T  A  T  T  E  R  S  F  S  A  M  N
     709
     Tip100 transposase
     Right recognition sequence (1513 bp)

        3610      3620      3630      3640      3650      3660      3670      3680      3690      3700      3710      3720      3730      3740      3750
3601 ATATAGTCAAGACTACACTTCGTAACAAGATGGAAGATGAATTTCTTAGTGATTGTTTGTTAGTATACATTGAAAAGCAAATTGCAAAACAATTTAGTATAGATTCAATTATAGATGCTTTTCGTGACATGCAAGAGAGACGGTCTAAAT 3750
      I  V  K  T  T  L  R  N  K  M  E  D  E  F  L  S  D  C  L  L  V  Y  I  E  K  Q  I  A  K  Q  F  S  I  D  S  I  I  D  A  F  R  D  M  Q  E  R  R  S  K  F
     759
     Tip100 transposase
     Right recognition sequence (1513 bp)

        3760      3770      3780      3790      3800      3810      3820      3830      3840      3850      3860      3870
3751 TTTAGTAGATTTGTAATATAATGATGTATTTTGGAACATGTTAAATGAATATTATTATATTTCAGTATTCTGTCTTAAATTGTGATTGGCCCCCCGCATTGGTTAATCCTGGCTCCGCCCCTG 3873
      *
     809
     Tip100 transposase      Right recognition sequence (100 bp)
     Right recognition sequence (1513 bp)
                                                                                                                  terminal inverted repeat
```
